# Modelling Relationships Between Extrusion Conditions and Quality Attributes of Expanded Snacks

**DOI:** 10.3390/foods15122118

**Published:** 2026-06-12

**Authors:** Danyang Ying

**Affiliations:** Agriculture & Food, Commonwealth Scientific and Industrial Research Organisation, 671 Sneydes Rd, Werribee, Melbourne, VIC 3030, Australia; danyang.ying@csiro.au

**Keywords:** expanded snacks, extrusion, response surface methodology, mixture-process model, mechanistic model, specific mechanical energy, die pressure, machine learning, digital twin, quality prediction

## Abstract

Expanded snack extrusion is governed by tightly coupled interactions among raw material composition, moisture, barrel temperature, screw speed, feed rate, screw configuration, die geometry, and energy input. These variables affect not only final responses such as expansion ratio, bulk density, hardness, crispness, and water absorption or solubility indices, but also intermediate state variables including specific mechanical energy (SME), melt temperature, die pressure, melt viscosity, and bubble growth dynamics. As a result, modelling has become essential for product design, process optimisation, and scale-up. This review critically evaluates the major classes of models used to describe process–structure–quality relationships in the extrusion of expanded snacks. The literature shows that empirical regression and response surface methodology (RSM) remain the most widely applied tools because they are experimentally efficient and easy to interpret. However, mixture-process designs are more appropriate when formulation and operating variables are changed simultaneously, while phenomenological and mechanistic approaches provide better physical insight into expansion and structure development. More recently, machine-learning and interpretable artificial intelligence approaches have demonstrated strong predictive capability when large, well-curated datasets are available. Across model families, a consistent theme is that operating variables act on final product quality through intermediate process state variables rather than independently. On that basis, this review proposes a practical hybrid framework for expanded snack extrusion: a mixture-process quadratic model augmented with SME, die pressure, melt temperature and shear-related state variables, and structured in three levels linking (i) controllable inputs to state variables, (ii) state variables to measurable quality attributes, and (iii) quality attributes to a gold-standard product target or sensory-control criterion. Such a model offers a realistic balance between predictive performance, physical interpretability, experimental burden, and industrial usefulness, while also providing a clear pathway toward future digital twin and machine-learning-enabled optimisation.

## 1. Introduction

Extrusion cooking is one of the most versatile thermo-mechanical processing operations used to produce expanded snacks, ready-to-eat cereals, crisp breads and structured foods. In a twin- or single-screw extruder, particulate or flour-based feed materials are transported, compacted, mixed, plasticised, gelatinised, denatured and partially melted within a short residence time [1,2,3,4,5]. Besides barrel temperature and feed moisture, shear stress and shear rate are critical processing factors because they determine mechanical energy dissipation, molecular breakdown, mixing intensity and matrix transformation. These shear-related variables are governed by screw-speed or screw-tip velocity, screw configuration, fill level, material viscosity and die geometry, including die diameter, land length and pressure drop [1,2,6,7]. For expanded snacks, the process is especially sensitive because quality depends not only on cooking but also on rapid die-exit expansion and setting.

The quality of an expanded snack is commonly described by expansion ratio, sectional expansion index, bulk density, true density, porosity, cell-size distribution, cell wall thickness, hardness, crispness, colour, WAI, WSI and sensory acceptability [8,9,10,11,12,13,14,15,16,17,18,19,20,21]. These responses are influenced by feed moisture, ingredient composition, particle size, barrel temperature, screw-speed or screw velocity, feed rate, screw configuration and die geometry. However, formulation can be as important as, and sometimes more important than, nominal extrusion parameters. The same temperature, moisture and screw-speed settings may produce a well-expanded snack for one starch- or cereal-based formulation but a dense or poorly expanded product for another formulation because starch type, amylose/amylopectin ratio, protein functionality, fibre level and particle size, lipid content, water-binding capacity, and starch damage alter melt viscosity, elasticity and bubble-wall stability [11,12,17,20,22,23]. This means that most empirical models are material- and formulation-specific unless they include transferable material descriptors or intermediate process state variables.

Predictive modelling is therefore central to rational product development. Empirical regression and RSM remain widely used because they are experimentally efficient and interpretable [8,9,10,11,12,13,14,15,16,17,18,19]. Mixture-process models are more suitable when ingredients and operating variables vary simultaneously [17,18,19,20,21,22,24]. Partial least squares regression and multivariate statistical approaches are useful when raw material composition and process variables are strongly correlated [17]. Mechanistic models, including analytical descriptions of flow, die pressure, heat transfer, bubble nucleation and bubble growth, provide physical insight but require material properties that are difficult to measure under extrusion conditions [6,7,25,26,27,28,29,30]. More recent work has introduced computational simulation, stochastic modelling, interpretable machine learning, active learning and digital-twin concepts [7,31,32,33,34,35,36,37,38].

The novelty of this review is the synthesis of existing modelling approaches into a state-variable-centred hierarchy proposed by the author. The term is used here as an organising framework rather than as a previously established standalone model class. It is supported by the extrusion literature showing that SME, die pressure, melt temperature, rheology, residence time, water activity and expansion dynamics mediate the relationship between set points and final quality [1,2,6,7,25,26,27,28,29,30,34,35,36,37,38]. Rather than treating moisture, temperature and screw speed as direct independent predictors of final quality, the review argues that they act through a smaller set of intermediate state variables: SME, die pressure, melt temperature, melt viscosity, shear stress/shear rate, residence time distribution, water activity/superheating and degree of material transformation. These states then control cellular structure formation and measurable quality. This perspective directly addresses the need for models that are interpretable, experimentally realistic and sufficiently transferable for industrial use.

The objectives are: (i) to clarify the quality attributes and measurement criteria used in expanded snack extrusion; (ii) to critically compare empirical, mixture-process, mechanistic, computational and data-driven model families; (iii) to integrate recent cross-disciplinary advances from rheology, foam science, CFD, finite element modelling, active learning and digital-twin research; and (iv) to propose an actionable hybrid framework for future snack extrusion modelling.

## 2. Quality Attributes and Measurement Criteria for Expanded Snacks

A comprehensive modelling review must first define the quality criteria to be modelled. Expanded snack quality is multidimensional and cannot be reduced to one response such as expansion ratio. Expansion and density describe macrostructure; porosity and cell-size distribution describe microstructure; hardness and crispness describe mechanical and acoustic texture; WAI and WSI describe starch and matrix transformation; and sensory acceptance integrates consumer perception [12,20,21,28,39] (Table 1).

Expansion ratio is typically calculated from the ratio of extrudate diameter to die diameter, or from sectional expansion when cross-sectional area is measured. Bulk density is calculated from mass and volume and is inversely related to expansion, although the relationship is not always linear because shrinkage and cell wall collapse can occur after die exit. Texture is usually quantified by compression, puncture, cutting or Kramer shear tests, while crispness can be better represented by combining force-displacement behaviour with acoustic emission or fracture-event analysis [21,39].

Functional indices such as WAI and WSI provide indirect evidence of starch gelatinisation, dextrinisation and molecular degradation. WAI reflects the ability of the processed matrix to absorb water, whereas WSI increases when soluble polysaccharides, dextrins or low-molecular-weight compounds are produced. For fibre- or protein-enriched snacks, additional measurements such as protein digestibility, phenolic retention, lipid oxidation, sensory attributes and tribological behaviour may be necessary [12,17,20,21].

For industrial or sensory-led optimisation, quality should also be linked to a recognised target or gold-standard control. This may be a benchmark commercial product, an internally approved reference product, a trained sensory-panel target, or a specification window for expansion, density, hardness, crispness and acceptability. Instrumental measurements are therefore best interpreted as predictors of whether the final product reaches this target, rather than as the target itself.

## 3. Process Variables, State Variables and Coupling Mechanisms

Expanded snack extrusion can be represented as a process–structure–property chain. Controllable inputs include formulation, particle size, feed moisture, feed rate, screw-speed or screw-tip velocity, barrel temperature profile, screw configuration, die land length, die diameter, die shape and downstream drying. These variables control a smaller set of state variables: SME, die pressure, melt temperature, residence time distribution, apparent viscosity, shear stress/shear rate, elastic recovery, water activity and degree of starch/protein transformation [1,8,18,26].

Feed moisture has a dual role. It plasticises starch/protein melts and reduces viscosity and SME, but it also modifies the thermodynamic driving force for flash evaporation at die exit. Too little moisture can cause excessive mechanical energy, scorching or poor flow; too much moisture can reduce superheating and melt strength, leading to low expansion and dense products [8,11,18]. Barrel temperature influences starch gelatinisation, protein denaturation, viscosity, vapour pressure and die-exit temperature. Screw speed affects shear rate, residence time, mechanical energy dissipation and mixing. Feed rate affects barrel fill and the ratio of mechanical energy to mass throughput. Die geometry imposes pressure drop, extensional flow and release conditions, making it central to expansion and shape stability [6,7,26].

Raw materials modify these state variables. Starch type, amylose content and starch damage affect gelatinisation and melt elasticity. Insoluble fibre can interrupt bubble walls and reduce expansion, while soluble fibre can increase viscosity or water binding. Protein can strengthen or disrupt the matrix depending on denaturation, aggregation and water competition. Lipid may reduce friction and SME and can weaken expansion by lubricating the melt [12,17,20,23]. Thus, a model fitted only to set points may fail when the ingredient lot or formulation changes.

## 4. Empirical Regression, RSM and Mixture-Process Models

Empirical regression and RSM are the most widely used modelling approaches in expanded snack development. A typical second-order RSM model relates a response Y to coded process variables X_i_ and their interaction and quadratic terms. It is efficient for local optimisation and provides interpretable coefficients, contour plots and desirability functions. Classic snack studies used RSM to link moisture, temperature, screw speed and ingredient level to expansion, density, hardness, WAI and WSI [8,9,10,11,18,19]. Recent studies continue to apply RSM for cereal-legume blends, functional snacks and fortified products [15,16,17,40,41,42].

However, RSM is a local statistical approximation. It is reliable only within the design space and does not necessarily explain why a response changes. A positive coefficient for temperature in one formulation may become negative in another if melt viscosity, moisture distribution or starch/protein ratio changes. Therefore, RSM should not be presented as a universal process model.

Mixture-process models should be treated as an extension of empirical/RSM modelling rather than a completely independent model family. Their key distinction is that ingredient proportions are constrained to sum to unity, while process variables remain independently adjustable. This is important for snack development because formulation and processing are usually co-optimised. For example, increasing fibre or protein content may reduce expansion, but higher temperature, lower moisture or modified screw speed may partially compensate. Mixture-process designs can identify such trade-offs more rigorously than conventional factorial designs [17,18,19,20,21,22].

Partial least squares regression provides another empirical option when predictors are correlated or numerous. Ramos Diaz et al. used PLSR to model corn-based snacks containing kañiwa and lupine, linking grain type, formulation, die temperature, screw speed, moisture and composition to sectional expansion, stiffness and nutritional properties [20]. Such approaches are useful when formulation descriptors and process variables are combined, but they also require careful validation.

## 5. Phenomenological, Mechanistic and Computational Models

Mechanistic models seek to describe extrusion and expansion using physical principles. They include descriptions of material flow, heat transfer, mechanical energy dissipation, die pressure, rheology, water phase change, bubble nucleation, bubble growth, coalescence, shrinkage and solidification. These models are essential for explaining why processing conditions affect quality and for extrapolating beyond a narrow experimental design [1,6,7,25,26,27,28,29,30].

In barrel and die flow models, the melt is commonly treated as a non-Newtonian, shear-thinning and temperature-dependent material. The apparent viscosity can be represented by a power-law or Herschel–Bulkley type relationship, often combined with Arrhenius- or WLF-type temperature and moisture dependencies (Table 2). Pressure generation depends on drag flow, pressure flow, screw geometry, fill, die resistance and material viscosity. Mixing theories and residence time distribution models are important because starch gelatinisation, protein denaturation and water redistribution depend on both intensity and duration of thermo-mechanical treatment.

Expansion at die exit can be interpreted as a foam formation problem. When the melt exits the die, pressure drops abruptly, superheated water flashes into vapour, and bubbles nucleate and grow. Bubble growth is driven by vapour pressure and dissolved gas expansion but resisted by viscosity, elasticity, surface tension and matrix setting. The critical bubble radius concept links nucleation to pressure difference and interfacial tension, while microcellular foam models describe the competition between expansion and solidification [25,27,28,42].

Die design is a mechanistic control point rather than a minor processing detail. Die land length, diameter, shape, wall slip, pressure drop and extensional flow history affect bubble initiation and the final shape of the extrudate [6,7,26]. Recent CFD-based work on die geometry and processing parameters reinforces that quality can be regulated by coupling die design with temperature, moisture and screw speed rather than optimising set points alone [7].

Computational approaches are expanding the mechanistic toolbox. Molecular dynamics and computational modelling have been used to explore protein–starch interactions at extrusion-relevant temperatures [31]. Finite element analysis is emerging in plant–protein texturisation and may inform future snack models where deformation, fracture or anisotropic structure are important [32]. Coupled heat–moisture–phase–change models from baking and puffing are also relevant because extrusion expansion involves rapid water phase change and large deformation [30,33]. Nevertheless, mechanistic models remain limited by the scarcity of reliable melt rheology, heat transfer, water activity and phase transition data under extrusion conditions.

## 6. Data-Driven, Machine-Learning and Digital-Twin-Oriented Models

Machine learning and artificial intelligence (AI) can capture nonlinear and high-order interactions that are difficult to specify in quadratic RSM. Neural networks, random forests, gradient boosting, Gaussian-process models, and Bayesian optimisation can be useful when sufficient, well-curated data exist. Recent work demonstrates active learning and robotics for low-moisture extrusion optimisation, interpretable machine learning for industrial extrusion diagnostics, and LLM-assisted dataset supplementation for extrusion data extraction [34,35,36]. A digital twin is a dynamic digital representation of a physical extrusion process that combines process data, sensor measurements, models, and updating algorithms to simulate, diagnose or optimise the behaviour of the real process. Digital-twin reviews in food processing therefore highlight the potential of combining sensors, simulation, AI and cyber-physical systems [37,38].

For expanded snacks, AI and machine learning should therefore be used as an upgrade path rather than a replacement for sound experimental design and mechanism. A key advantage is that AI may reveal nonlinear relationships among inputs, state variables and final product attributes that are not evident from individual measurements or simple two-factor plots. However, the model still needs a reliable target: a gold-standard product, validated sensory-control sample, consumer-acceptance criterion or industrial specification against which optimisation can be judged. Minimum requirements include standardised input metadata, batch-level traceability, external validation, uncertainty quantification, feature importance or counterfactual analysis, and explicit reporting of the domain over which the model is valid. For small datasets, interpretable regression, PLSR or Gaussian-process models may be preferable to deep learning. For large industrial datasets, hybrid AI models can be structured around state variables to improve transferability and enable control of both inputs and intermediate states toward the desired final product.

## 7. Comparative Assessment of Model Families

A clearer taxonomy is needed to avoid comparing non-equivalent model classes. In this review, model families (Table 3) are grouped as: (i) empirical/statistical models, including regression, RSM, mixture-process designs and PLSR; (ii) phenomenological and mechanistic models; (iii) computational physics models, including CFD, finite element and stochastic simulations; (iv) data-driven and machine-learning models; and (v) hybrid state-variable models. Mixture-process models are therefore not treated as a fully independent category alongside RSM, but as a specialised empirical design structure for constrained ingredient systems.

## 8. Recommended Hybrid State-Variable Framework

The most practical framework for expanded snack extrusion is a hybrid three-level model. Level 1 relates controllable inputs to process state variables. Level 2 relates state variables and selected formulation descriptors to measurable quality attributes. Level 3 relates those quality attributes to a final product target or gold-standard control, such as a benchmark product, trained sensory reference, consumer-acceptance threshold or industrial specification. This directly reflects the process-structure-quality-target chain and provides a route for troubleshooting. If bulk density increases, the model can indicate whether the likely cause is insufficient SME, excessive moisture, reduced die pressure, low melt temperature or weak bubble-wall setting, and whether the change is large enough to move the product outside the accepted target range.

Level 1 can be represented as S_j_ = f_j_(X, M, XxM, X^2^, M^2^), where S_j_ includes SME, die pressure, melt temperature, apparent viscosity, shear stress/shear rate, residence time or water activity; X contains process variables; and M contains mixture components or formulation descriptors. This equation can have the same quadratic structure as a conventional RSM model. Its specific role here is to predict intermediate states rather than final quality directly. Level 2 can be represented as Y_k_ = g_k_(S, X, M), where Y_k_ includes expansion, density, hardness, crispness, WAI, WSI or sensory scores. Level 3 can be represented as Q = h(Y, G), where Q is the product acceptability or conformity score and G is the gold-standard target or control. For small studies, Levels 1 and 2 may be quadratic or PLSR models and Level 3 may be a desirability function or specification window. For larger industrial datasets, Level 1 can incorporate first-principles calculations and Levels 2–3 can incorporate interpretable AI.

A practical example would be a fibre-enriched corn snack. In a conventional RSM, fibre level, moisture and temperature are fitted directly to expansion and hardness. In the proposed framework, fibre level, moisture, temperature, screw-speed or screw-tip velocity and die geometry first predict SME, die pressure, melt temperature and shear-related state variables. These state variables then predict sectional expansion, density, fracture force and crispness. Finally, these quality attributes are compared with a reference expanded snack or sensory-control target to decide whether the product is acceptable. If fibre reduces expansion because it lowers melt elasticity and disrupts cell walls, this should appear through changes in pressure, viscosity proxy variables, shear-related variables and structure descriptors rather than only as a negative fibre coefficient.

Error propagation should be considered explicitly. Uncertainty in torque measurement affects SME; uncertainty in pressure transducers affects die-pressure estimates; and image-analysis segmentation affects porosity and cell-size descriptors. Therefore, future studies should report measurement uncertainty, replicate variability and confidence intervals. When Level 1 errors are large, Level 2 predictions should include propagated uncertainty rather than single deterministic values.

## 9. Implications for Experimental Design, Scale-Up and Digital Twins

Experimental design should match data maturity. For exploratory work with 20–40 runs, a central composite, Box–Behnken or D-optimal mixture–process design remains appropriate. For formulation studies, constrained mixture-process designs are preferable. For mechanistic or hybrid studies, experiments should include targeted measurements of torque, die pressure, melt temperature, residence time distribution, rheology or structural descriptors only when these measurements are needed to explain mechanisms or improve transferability. Adding extensive physicochemical characterisation changes the model from a purely empirical model toward a phenomenological, mechanistic or hybrid model; this is appropriate only when the research objective is to build such a model. For industrial datasets, batches should be split for validation by time, formulation or extruder rather than random record splitting.

A digital twin for expanded snacks should be developed progressively. Stage 1 is a curated dataset with standardised quality measurements and a defined gold-standard product or specification. Stage 2 is a calibrated RSM or mixture-process model. Stage 3 adds state variables and mechanistic calculations. Stage 4 introduces inline sensors, uncertainty estimation and interpretable AI. Stage 5 closes the loop with optimisation or model predictive control, allowing the system to adjust inputs and intermediate states toward the final product target. This staged pathway is more realistic than attempting to build a complete physics-based digital twin from the outset.

## 10. Research Gaps and Future Directions

Several targeted gaps remain. First, most published snack extrusion studies are still local optimisation studies, with narrow ingredient systems and insufficient external validation. Second, quality measurement is not standardised; expansion, density, texture and crispness are often measured by different protocols, preventing reliable cross-study modelling. Third, melt rheology and water phase behaviour under actual extrusion moisture, temperature and shear conditions remain poorly quantified. Fourth, die-exit expansion models rarely integrate die geometry, pressure drop, bubble nucleation, bubble growth, shrinkage and wall setting in one experimentally validated framework.

Future work should therefore prioritise: (i) shared reporting templates for formulation, extruder configuration, process variables, state variables and quality measurements; (ii) standardised measurement of expansion, density, hardness, crispness and cell structure; (iii) clear definition of gold-standard targets or sensory-control products for optimisation; (iv) rheological and thermodynamic data for starch-, fibre- and protein-rich melts under extrusion-relevant conditions; (v) mechanistic studies linking die design to bubble growth and cellular architecture; (vi) hybrid models that include state variables and uncertainty propagation; (vii) external validation across extruders and ingredient lots; and (viii) interpretable AI tools that can discover hidden input–state–quality relationships while remaining constrained by validated product targets and process understanding.

## 11. Conclusions

The central conclusion is that extrusion set points influence final snack quality through intermediate state variables. SME, die pressure, melt temperature, apparent viscosity, shear stress/shear rate, and residence time distribution provide a more transferable bridge between raw material/process inputs and final quality attributes. A hybrid three-level state-variable and product-target framework is therefore recommended as the most practical pathway for future modelling. Such a framework can support experimental design, troubleshooting, scale-up, AI-enabled optimisation, digital-twin development and ultimately more rational design of expanded snacks with targeted structure, texture, nutrition, sensory quality and product acceptability.

## Figures and Tables

**Table 1:** Quality attributes and measurement criteria for expanded snacks.

Quality Attribute	Typical Measurement	Modelling Relevance	Main Limitations
Expansion ratio/sectional expansion index	Diameter or cross-sectional area relative to die opening	Primary indicator of puffing and die-exit bubble growth	Sensitive to product shape, shrinkage and non-circular cross-sections
Bulk density	Mass divided by bulk volume	Industrial proxy for lightness, bowl life and packaging density	Affected by cutting, irregular shape and void distribution
Porosity/cell-size distribution	Image analysis, microscopy or X-ray tomography	Links bubble nucleation/growth to mechanical texture	Requires standard segmentation and representative sampling
Hardness/breaking force	Compression, puncture, three-point bending or Kramer shear	Relates to bite force and matrix strength	Method-dependent and affected by sample orientation
Crispness	Mechanical–acoustic tests and sensory panels	Captures fracture events and perceived crispness	Needs acoustic calibration and sensory validation
WAI/WSI	Hydration and centrifugation protocols	Indicates starch transformation and molecular degradation	Protocol-dependent and not specific to one mechanism
Colour and sensory acceptance	Instrumental colour and trained/consumer panels	Important for product optimisation and desirability functions	Sensory data are expensive and context-dependent

**Table 2:** Common concepts, definitions and variables used in modelling of extruded snacks.

Equation/Concept	Representative Form	Variables	Use in Snack Extrusion
Specific mechanical energy	SME = (2π N T)/(m_dot)	N: screw speed; T: torque; m_dot: mass flow rate	Captures mechanical energy input per unit mass and links screw speed, viscosity and feed rate to transformation
Power-law viscosity	η_app_ = K γ_dot^(n−1)^	K: consistency; γ_dot: shear rate; n: flow index	Represents shear-thinning starch/protein melts in barrel and die flow models
Temperature dependence	K = K_0_ exp(E_a_/RT)	Ea: activation energy; R: gas constant; T: absolute temperature	Describes reduction in viscosity with increasing melt temperature
Moisture plasticisation	K = K_0_ exp(−aM)	M: moisture content; a: empirical coefficient	Represents viscosity reduction and SME reduction with increasing moisture
Laplace pressure	ΔP = 2σ/r	σ: surface tension; r: bubble radius	Defines pressure needed to stabilise or grow a bubble
Critical bubble radius	r_c_ = 2σ/(P_v_ − P_m_)	P_v_: vapour/gas pressure; P_m_: matrix pressure	Links nucleation to pressure drop and interfacial tension
Expansion ratio	ER = D_e_/D_d_ or A_e_/A_d_	D/A: extrudate and die diameter/area	Primary final response for puffing and shape development
Bulk density	ρ_b_ = m/V_b_	m: sample mass; V_b_: bulk volume	Quality response linked to expansion, porosity and packaging
Two-level state-variable model	S_j_ = f(X, M); Y_k_ = g(S, X, M)	S: state variables; X: process; M: mixture	Separates input-to-state and state-to-quality relationships

**Table 3:** Common model families, their strengths and limitations for the extruded snacks.

Model Family	Strengths	Limitations	Best Use Case	Key Validation Requirement
Regression/RSM	Efficient, interpretable, low experimental burden	Local validity, weak extrapolation, limited mechanism	Initial optimisation of moisture, temperature, screw speed and feed rate	Replicated centre points and independent confirmation runs
Mixture-process designs	Handles constrained formulations and process interactions	More complex design and interpretation	Co-optimisation of ingredient proportions and operating conditions	Validation at optimum and at formulation boundaries
PLSR/multivariate regression	Handles correlated predictors and composition data	May be difficult to interpret mechanistically	Combining compositional descriptors with process variables	Cross-validation and external batch validation
Mechanistic/analytical models	Physical insight and potential transferability	Requires difficult-to-measure material properties	Understanding rheology, die pressure, bubble growth and scale-up	Independent rheology/pressure/temperature measurements
CFD/FE/stochastic models	Spatial, dynamic and uncertainty-aware predictions	High computational and data demands	Die design, flow balance, deformation and variability analysis	Comparison with measured pressure, temperature and product structure
Machine learning	Captures nonlinear interactions and large datasets	Overfitting, poor generalisation and interpretability risk	Industrial datasets, sensor-rich operations, optimisation	External validation, leakage control and uncertainty reporting
Hybrid state-variable models	Balances interpretability, feasibility and predictive power	Requires state-variable measurement and staged calibration	Pilot-to-industrial modelling and digital-twin development	Separate validation of input-to-state and state-to-quality models

## Data Availability

No new data were created or analysed in this study.

## Abbreviations

The following abbreviations are used in this manuscript:

**Abbreviation**	**Definition**
CFD	Computational fluid dynamics
ER	Expansion ratio
FE	Finite element
ML	Machine learning
PLSR	Partial least squares regression
RSM	Response surface methodology
SME	Specific mechanical energy
WAI	Water absorption index
WSI	Water solubility index
